# Pod5Viewer: a GUI for inspecting raw nanopore sequencing data

**DOI:** 10.1093/bioinformatics/btae665

**Published:** 2024-12-03

**Authors:** Vincent Dietrich, Nicolò Alagna, Mark Helm, Susanne Gerber, Tamer Butto

**Affiliations:** Institute of Human Genetics, University Medical Center of the Johannes Gutenberg University Mainz, Mainz, 55128, Germany; Institute of Human Genetics, University Medical Center of the Johannes Gutenberg University Mainz, Mainz, 55128, Germany; Institute of Pharmaceutical and Biomedical Sciences, Johannes Gutenberg-University Mainz, Mainz, 55128, Germany; Institute of Human Genetics, University Medical Center of the Johannes Gutenberg University Mainz, Mainz, 55128, Germany; Institute for Quantitative and Computational Biosciences (IQCB), Johannes-von-Müller-Weg 6, Mainz, 55128, Germany; Institute of Pharmaceutical and Biomedical Sciences, Johannes Gutenberg-University Mainz, Mainz, 55128, Germany

## Abstract

**Motivation:**

Oxford Nanopore Technologies recently adopted the POD5 file format for storing raw nanopore sequencing data. The information stored in these files provides detailed insights into the sequencing features and enhances the understanding of raw nanopore data. However, the process of visualizing the data can be cumbersome, especially for users without programming skills. To address this issue, we developed the pod5Viewer, a GUI application for inspecting POD5 files.

**Results:**

The pod5Viewer offers straightforward access to raw sequencing data and associated metadata in POD5 files. It includes functionalities for viewing, plotting, and exporting individual reads. Designed with user-friendliness in mind, the pod5Viewer is easy to install and use, making it suitable for users with all technical backgrounds.

**Availability and implementation:**

The pod5Viewer is available as open source from the pod5Viewer Github repository (https://github.com/dietvin/pod5Viewer)

## 1 Introduction

During Nanopore sequencing, a DNA or RNA molecule passes through a transmembrane nanopore while a voltage is applied across the membrane. This causes the ionic current to be disrupted in a way specific to the nucleotides present in the pore at a given moment. These current signals are captured and transformed into corresponding nucleotide sequences during base-calling (Wang *et al.* 2021).

Initially, in the approach established by Oxford Nanopore Technologies (ONT), raw current signals and corresponding metadata were stored in the HDF5-based FAST5 format. However, with faster processing time and increased throughput coming hand in hand with improved chemistries and base-calling software, the relatively slow input/output speed of the FAST5 format turned into a potential bottleneck especially if users intend to employ the real-time analysis functions (Wierczeiko *et al.* 2023, Butto *et al.* 2024). These issues were first addressed with the community-developed SLOW5 format, which particularly improved parallel access (Gamaarachchi *et al.* 2022). Recently, ONT have released the POD5 format as the official replacement for the FAST5 format. POD5 optimizes the Apache Arrow framework for nanopore sequencing data, resulting in faster write and read speed and more efficient data storage compared to the FAST5 format. While the extent of these improvements is yet to undergo a systematic benchmark, the official adoption of POD5 as the go-to data format is increasingly leading to its wide usage, with all of the latest tools developed by ONT optimized for using POD5 files.

All mentioned formats come with a dedicated *C* or *Python*-based application programming interface (API) for accessing and manipulating respective files. While these provide in-depth functionalities, their usage through scripts and the command line can be cumbersome—especially for users without extensive programming knowledge. Easy access to the data has various advantages, like getting an overview of the raw current signal and the metadata, including the exact software and hardware used to generate it, enabling a more detailed understanding of the general characteristics of nanopore sequencing data. While the HDFView software (https://www.hdfgroup.org/downloads/hdfview/) provided just that in a graphical user interface (GUI) for the FAST5 format, comparable software is unavailable for the POD5 format. Given the official adoption by ONT, such an application can prove helpful for an increasing number of users. To this end, we developed the pod5Viewer, a lightweight GUI application for accessing and inspecting POD5 files. Similar to HDFView for FAST5 files, it provides a straightforward way of viewing the nested structure of POD5 files and the information stored within them. Beyond this, the pod5Viewer features plotting functionalities for the raw current signal stored in the files, as well as options for exporting contained features to a better readable format. The pod5Viewer was developed with ease of use in mind during both the installation and execution. It is openly available under a GPL-3.0 license.

## 2 Methods

### Installation and dependencies

The user has multiple options for installing the pod5Viewer. For Windows users, an installer is available, providing a streamlined installation and subsequent access for a convenient user experience. Similarly, for Ubuntu a DEB file is available for installation via apt. Alternatively, it can be installed from the Python package index via the pip command “*pip install pod5Viewer*” on all operating systems. All files are available from the pod5Viewer Github repository, along with detailed information for all install options (https://github.com/dietvin/pod5Viewer).

The GUI of the pod5Viewer is built in the Qt framework with the PySide6 package (v.6.5.2). Interactions with POD5 files are handled using the POD5 API provided by ONT (v.0.3.10) (see Supplementary data S1.3.1), and matplotlib (v.3.9.2) enables signal plotting functionalities (see Supplementary data S1.3.2).

### Usage

**Figure 1.**
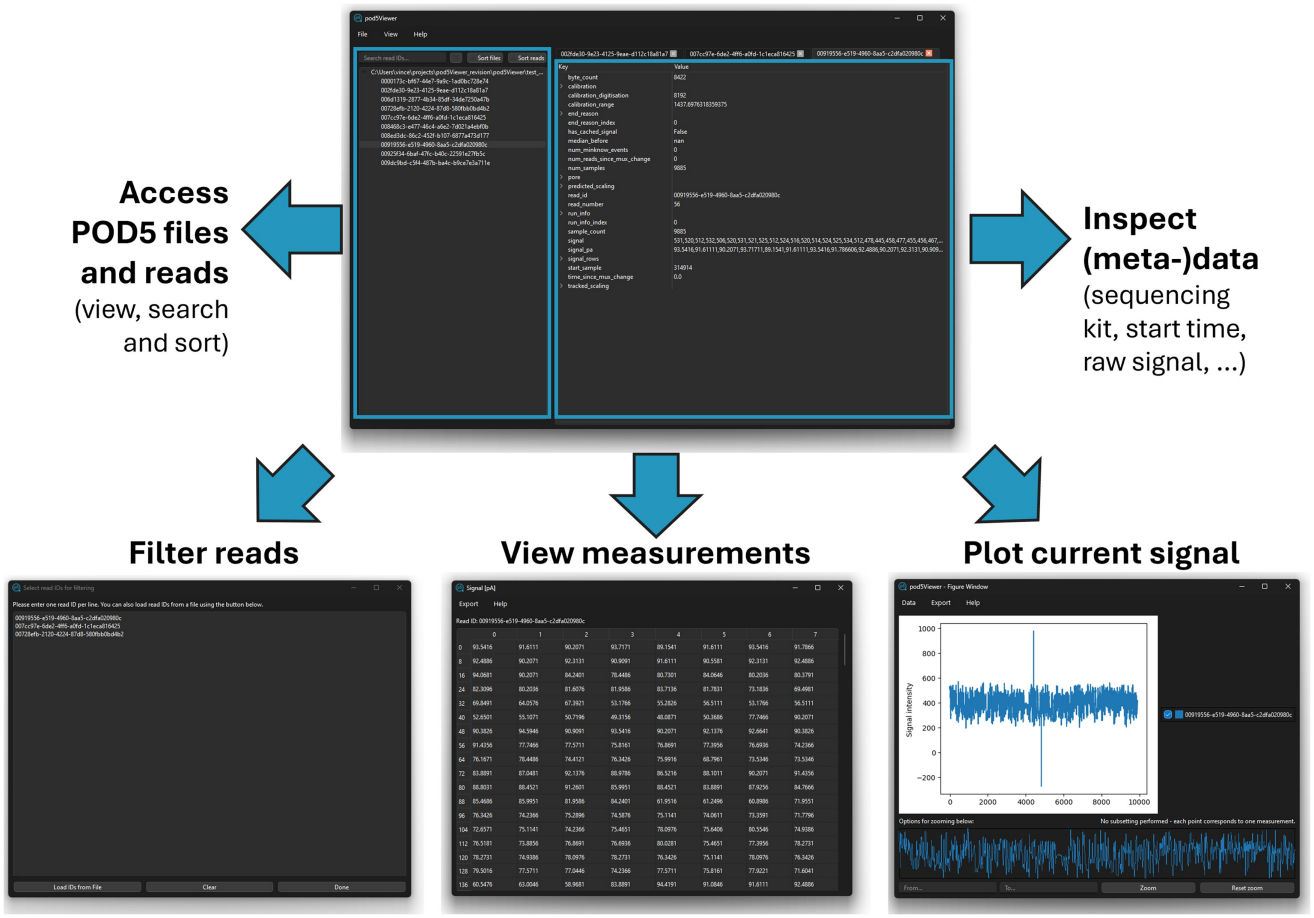
Overview of the pod5Viewer and its main features. Top: Main window containing the navigator (left) and data view panel (right). Bottom left: Read filtering to show only a specific subset of reads. Bottom center: Data viewing function showing individual measurements. Bottom right: Plotting function for visualizing the current signals for opened reads.

After starting the pod5Viewer, the user can load either one or multiple POD5 files into the application. Loaded files are listed on the left side of the window in the *file navigator panel* (Fig. 1, top left). The entry for each file can be expanded to reveal all reads stored within it, indicated by the read IDs. For straightforward file navigation, functions for filtering, searching, and sorting loaded reads are available (Fig. 1, bottom left; see Supplementary data S1.2.2 & S1.2.3).

Each read can be opened by clicking on the entry in the navigator panel. This opens a tab in the *data view panel* on the right side of the window (Fig. 1, top right). Here, all opened reads are given a tab. The data of the focused read are displayed as key–value pairs. As POD5 files contain nested information, these nested groups can be expanded in the data view panel by clicking the overarching entry, allowing for inspection of all contained information (see Supplementary data S1.2.4).

Viewing thousands of current measurements from the raw current signal simultaneously would lead to unresponsive application behavior. As such, the data view panel only contains a small subset of the raw current data. The entirety of the data can be accessed through the *View signal* function of the pod5Viewer, which displays all measurements of a given read in chunks in a separate window to retain fast performance of the application (Fig. 1, bottom center; see Supplementary data S1.2.5).

In addition to displaying measured values in numerical form, the pod5Viewer also provides several plotting functions, optionally calibrated to picoampere, as well as the option of plotting normalized signals. Signals of either all opened reads or only the currently focused one can be visualized in a separate window using the different *Plot signal* functions. The window contains an interactive plot that allows the user to zoom in to a signal interval of interest. The figure can also be exported to a SVG file (Fig. 1, bottom right; see Supplementary data S1.2.6).

Additionally, the pod5Viewer offers export options for either all information or only the signal measurements. Similar to the plotting functions, information from either all opened reads or only the currently focused read can be exported to different file formats, including JSON and binary Numpy format (see Supplementary data S1.2.7). This enables easier access to reads of interest in further downstream processing steps, as the user can directly access the data in a human-readable format without requiring the pod5 API.

Notably, all actions in the pod5Viewer have a keyboard shortcut assigned, so selection, navigation, and all other viewing and export options can be performed without the mouse for faster and more accessible usage (see Supplementary data S1.2.9).

More detailed instructions for the usage of the pod5Viewer are given in the Supplementary data (S1.2), as well as details about the backend for accessing, viewing, and plotting data (Supplementary data S1.3).

## 3 Discussion

Nanopore sequencing technology is continuously maturing with new chemistry and software for increasingly accurate and high-yield sequencing data. Adopting the POD5 format for raw sequencing data from these devices underscores this progress. In line with the HDF view software for the meanwhile deprecated FAST5 format, the here-introduced pod5Viewer makes the POD5 format easily accessible without the need for direct interaction with the API. It provides a lightweight, fast, and easy-to-navigate graphical user interface for a more thorough understanding of the general structure of the format and enables detailed inspection of the metadata and signals stored within it. This facilitates the identification of metadata relevant to correct base-calling and beyond for better planning of processing steps after sequencing (see Supplementary data S1.4.1) and allows for the identification of possible aberrations in the signal through the plotting functionalities (see Supplementary data S1.4.2). Additionally, the export options support further exploration and manipulation of raw nanopore sequencing data through straightforward access (see Supplementary data S1.4.3). In line with the ease of navigation within the application, the provided installation options ensure easy access to users with limited knowledge of programming and command line interfaces.

## Supplementary Material

btae665_Supplementary_Data
