# Supplementary material for: Pod5Viewer: a GUI for inspecting raw nanopore sequencing data
Source: Bioinformatics. 2024 Dec 3;40(12):btae665. doi: 10.1093/bioinformatics/btae665 (PMC11630908; doi:10.1093/bioinformatics/btae665)
Supplement: btae665_Supplementary_Data [file btae665_supplementary_data.pdf]

Supplementary information

Contents

1 Supplementary information 1
1.1 Installation 2
1.1.1 Windows 2
1.1.2 Linux 2
1.1.3 OS-independent 2
1.2 Detailed usage 3
1.2.1 Open file(s) 3
1.2.2 File Navigation 3
1.2.3 Read filtering 4
1.2.4 Open read(s) 5
1.2.5 View the current signal 7
1.2.6 Plot current signal 7
1.2.7 Export functions 8
1.2.8 Clear window 9
1.2.9 Shortcuts 10
1.3 Backend details 11
1.3.1 Communication with the pod5 API 11
1.3.2 Details on the plotting functions 11
1.4 Possible applications 12
1.4.1 General data exploration 12
1.4.2 Signal plotting 13
1.4.3 Data export 14
1.5 Test data 15

1.1 Installation

All files needed for the installation on a given operating system (OS) can be found on the latest release page in the pod5Viewer Github repository. (https://github.com/dietvin/pod5Viewer/releases/latest)

1.1.1 Windows

The pod5Viewer can be installed on Windows 11 or Windows 10 systems through the following steps:

1. Download the latest installer EXE file.
2. Start the installer and follow the instructions.
3. Run the pod5Viewer from the start menu.

Note that at the moment the pod5Viewer software is not known to the Windows defender. As such a Window titled *Windows protected your PC* might open when first starting the installer. To continue with the installation, the user needs to press *More Info* and *Run anyway*.

1.1.2 Linux

On Ubuntu and Linux Mint the installation goes as follows:

1. Download the DEB file for a given version of the operating system.
2. Open the terminal and navigate to the folder containing the downloaded file
3. Run apt to install the package:
   ```
   sudo apt install -y ./pod5viewer_<version>.deb
   ```
4. Run the pod5Viewer from the start menu or the terminal by typing `pod5Viewer`

1.1.3 OS-independent

The pod5Viewer can also be installed on any system running Python via the Python Packaging Index. Here it is also highly recommended to install the pod5Viewer into a virtual environment via Conda or comparable solutions. This can be done from a terminal using the following commands:

```
conda create -n p5 python==3.11

conda activate p5

pip install pod5Viewer
```

Note that when installing it this way the pod5Viewer can only be started from the command line by typing `pod5Viewer` and POD5 files on the system do not get associated to the application. As such it is not possible to directly open a file by clicking on it in the desktop environment.

1.2 Detailed usage

1.2.1 Open file(s)

When the pod5Viewer is opened, files can be loaded via File > Open File(s)… or Open directory… (Figure 1, top left). The latter opens all POD5 files contained in a given directory. On Linux or other OS after installation via pip, files can be opened from the command line by typing pod5Viewer, followed by the path to one or more POD5 files (Figure 1, center left). Alternatively, when the application was installed via the Windows installer or apt on Linux, files can be selected and opened from the file browser or the desktop (Figure 1, bottom left). After opening one or multiple files, the file path is shown in the navigator panel on the left side of the window (Figure 1, right).

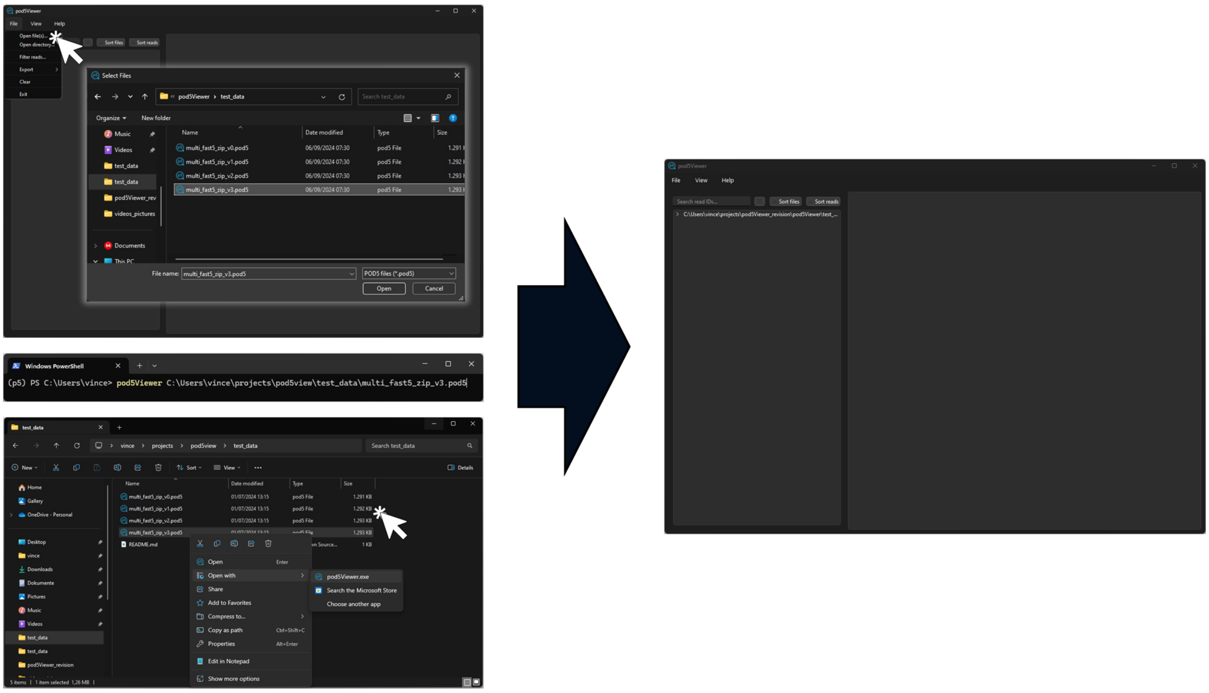

Figure 1 Left: Options for opening files with the pod5Viewer by selecting a file from the pod5Viewer, opening it from the terminal, or opening it from the file browser. Right: View directly after loading a file.

1.2.2 File Navigation

Once the path of an opened file is shown in the navigator panel, contained reads can be shown by expanding the view through double clicking on the file path (Figure 2). The search bar above the file list allows for searching a specific read ID in the file(s). Two sort buttons allow for sorting listed files and reads independently from each other, in either ascending or descending order (Figure 2, right).

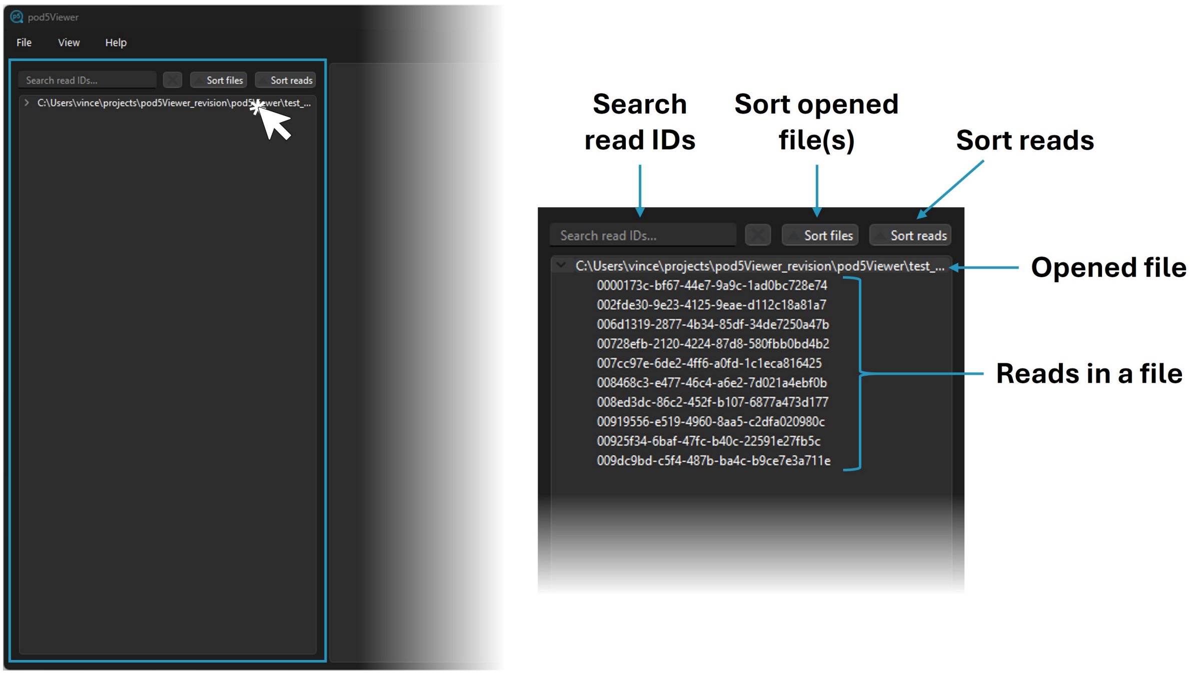

Figure 2 Overview of the options for file navigation. Left: File navigator panel (framed in blue) with collapsed file entry. Right: Zoomed in view of the same file after expanding it with explanations of the navigation options.

1.2.3 Read filtering

Loaded reads can be filtered by a given subset of read IDs (Figure 3, left). This feature can be accessed via File > Filter reads…. This opens a separate window containing a text browser. Here read IDs of interest can be provided, either by typing them manually, pasting them or loading them from a file via the Load IDs from File button. A selection of IDs can be submitted via the Done button. Afterwards the file navigator panel shows only reads that are found in the given selection (Figure 3, right). Active filtering can be reverted by reopening the window, pressing the Clear button and submitting again.

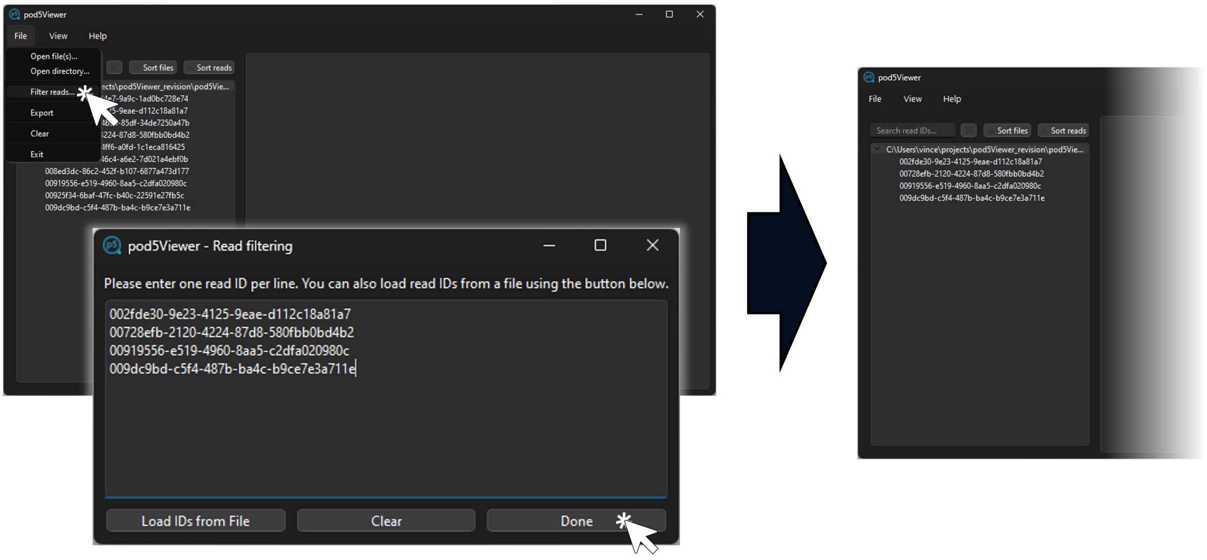

Figure 3 Overview of read filtering. Left: Window for read ID input opened through the “File > Filter reads…” menu. Right: View of the file navigator panel after applying the filter.

1.2.4 Open read(s)

To open individual reads, a read ID can be selected in the navigator panel (Figure 4). A single click opens the read information as a preview. A preview tab will be overwritten once another read is selected. Each read that should remain open must be double-clicked to be opened as a permanent tab. Once opened permanently, it remains in the background if another read is selected, allowing the user to open multiple reads at once. A permanent tab can be closed by pressing the X symbol on a tab. Each tab contains all information stored in the entry of a given read in a nested key-value pair structure. Overarching groups can be expanded and values can be selected by double-clicking on them, allowing them to be copied to the clipboard via the Ctrl+C key combination.

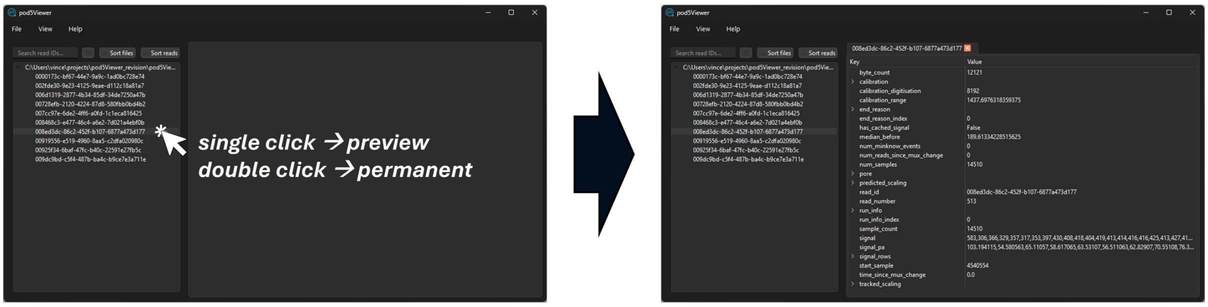

Figure 4 Options for viewing reads from opened POD5 files. Left: Empty data view panel with indications on how to open an entry. Right: Opened entry in the data view panel.

Hovering over a given key shows a short explanation. The explanations are collected from the POD5 documentation[1]. All included docstrings are shown in Table 1.

Table 1 Keys associated with the POD5 format and corresponding docstrings. Docstrings are collected from the POD5 documentation

Key	Explanation
byte_count	Number of bytes used to store the reads data
calibration	Calibration data associated with the read
calibration offset	Calibration offset used to convert raw ADC data into pA readings
calibration scale	Calibration scale factor used to convert raw ADC data into pA readings
calibration_digitisation	Digitisation value used by the sequencer. Intended to assist workflows ported from legacy file formats
calibration_range	Calibration range value. Intended to assist workflows ported from legacy file formats
end_reason	End reason data associated with the read
end_reason forced	True if it is a 'forced' read break (e.g. mux_change, unblock), false otherwise
end_reason name	Reason name as a lower string
end_reason reason	End reason enumeration
end_reason reason name	End reason enumeration name
end_reason reason value	End reason enumeration value
end_reason_index	Dictionary index of the end reason data associated with the read. This property is the same as the EndReason enumeration value
has_cached_signal	Cached signal is available for this read

[1] https://pod5-file-format.readthedocs.io/en/latest/

median_before	Get the median before level (in pico amps) for the read
num_minknow_events	Find the number of minknow events in the read
num_reads_since_mux_change	Number of selected reads since the last mux change on this reads channel
num_samples	Get the number of samples in the reads signal data
pore	Pore data associated with the read
pore channel	1-indexed channel,
pore pore_type	Name of the pore type present in the well,
pore well	1-indexed well
predicted_scaling	Predicted scaling value in the read
predicted_scaling scale	Scale of the predicted scaling
predicted_scaling shift	Shift of the predicted scaling
read_id	Unique read identifier for the read as a UUID
read_number	Get the integer read number of the read
run_info	Run info data associated with the read
acquisition_id	A unique identifier for the acquisition
acquisition_start_time	Clock time for sample 0
adc_max	The maximum ADC value that might be encountered
adc_min	The minimum ADC value that might be encountered
context_tags	The context tags for the run. (For compatibility with fast5)
context_tags barcoding_enabled	Barcoding is enabled
context_tags basecall_config_filename	Name of the config file used for basecalling
context_tags experiment_type	Type of experiment
context_tags sample_frequency	Sample frequency
context_tags selected_speed_bases_per_second	Selected speed in bases per second
context_tags sequencing_kit	Sequencing kit
experiment_name	User-supplied name for the experiment being run
flow_cell_id	Uniquely identifies the flow cell the data was captured on
flow_cell_product_code	Type of flow cell the data was captured on
protocol_name	Name of the protocol that was run
protocol_run_id	Unique identifier for the protocol run that produced this data
protocol_start_time	When the protocol that the acquisition was part of started
sample_id	User-supplied name for the sample being analysed
sample_rate	Number of samples acquired each second on each channel
sequencer_position	Sequencer position the data was collected on
sequencer_position_type	Type of sequencing hardware the data was collected on
sequencing_kit	Type of sequencing kit used to prepare the sample
software	Software that acquired the data
system_name	Name of the system the data was collected on
system_type	Type of system the data was collected on
tracking_id	Tracking id for the run. (For compatibility with fast5
run_info_index	Dictionary index of the run info data associated with the read
sample_count	Number of samples in the reads signal data
signal	Full signal for the read
signal_pa	Full signal for the read, calibrated in pico amp

signal_rows	All signal rows for the read
signal_rows batch_index	Alias for field number 0
signal_rows batch_row_index	Alias for field number 1
signal_rows byte_count	Alias for field number 3
signal_rows sample_count	Alias for field number 2
start_sample	Absolute sample which the read started
time_since_mux_change	Time in seconds since the last mux change on this reads channel
tracked_scaling	Tracked scaling value in the read
tracked_scaling scale	Scale of the predicted scaling
tracked_scaling shift	Shift of the predicted scaling

1.2.5 View the current signal

The measurement data can be viewed in its entirety through View > View signal… > View full signal data… or View full signal pA data… (Figure 5, left). This opens a new window that shows the data in chunks to provide a fast and responsive experience. Each chunk shows individual current measurements in sequence from top left to bottom right (Figure 5, right). The user can scroll through different chunks to see all measurements for a given read.

From the view window, measurements can be exported isolated from the remaining metadata. This feature can be accessed via the Export menu in the top of the window. Available formats are binary Numpy format[2] for later import into Numpy Python environments and human-readable text format.

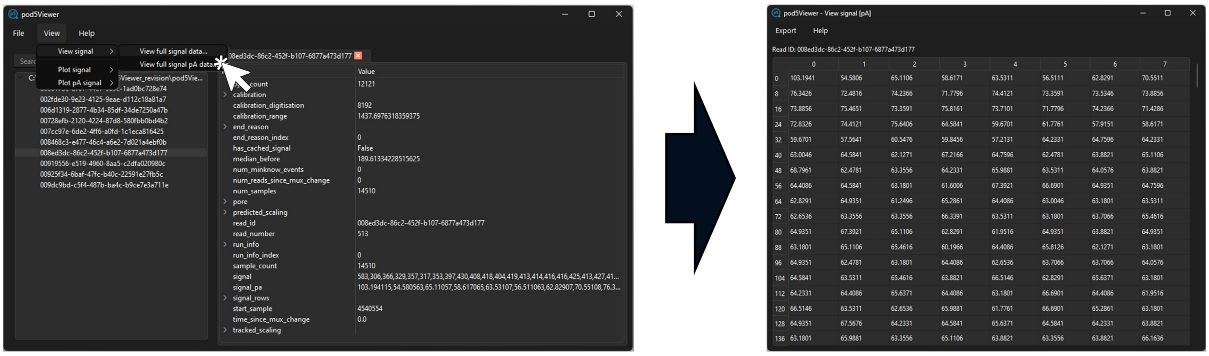

Figure 5 Viewing current measurements in their entirety. Left: Steps to take for opening the data view window. Right: Opened data view window.

1.2.6 Plot current signal

The signal can be visualized through View > Plot signal… or View > Plot pA signal… (Figure 6, left). Both menus contain a selection between Focussed read… and All opened reads….Upon a given selection a new window opens containing a line plot of the measurements for each shown read (Figure 6, right).

[2] https://numpy.org/doc/stable/reference/generated/numpy.lib.format.html#module-numpy.lib.format

Specific regions can be viewed by selecting a range with the mouse in the preview map below the plot. For this the user must press the left mouse button on the start point in the preview map, drag the mouse to the end point and release the left mouse button again. Upon a given selection, the plot updates to show only the selected region. Alternatively for a more precise selection, it is possible to type a start (From...) and end (To...) index into the input field below and confirm the choice by pressing the Zoom button. The Reset zoom button resets the figure to the initial state.

Signals of individual reads can be hidden by unchecking the corresponding label in the legend next to the plot. The user can switch between plotting normalized and non-normalized signals through the Data menu. A current view can be exported to SVG format through the Export menu.

Above the preview map, the text label on the right side tells the user if subsetting is active and if so, to what extent (see S1.3.2.2).

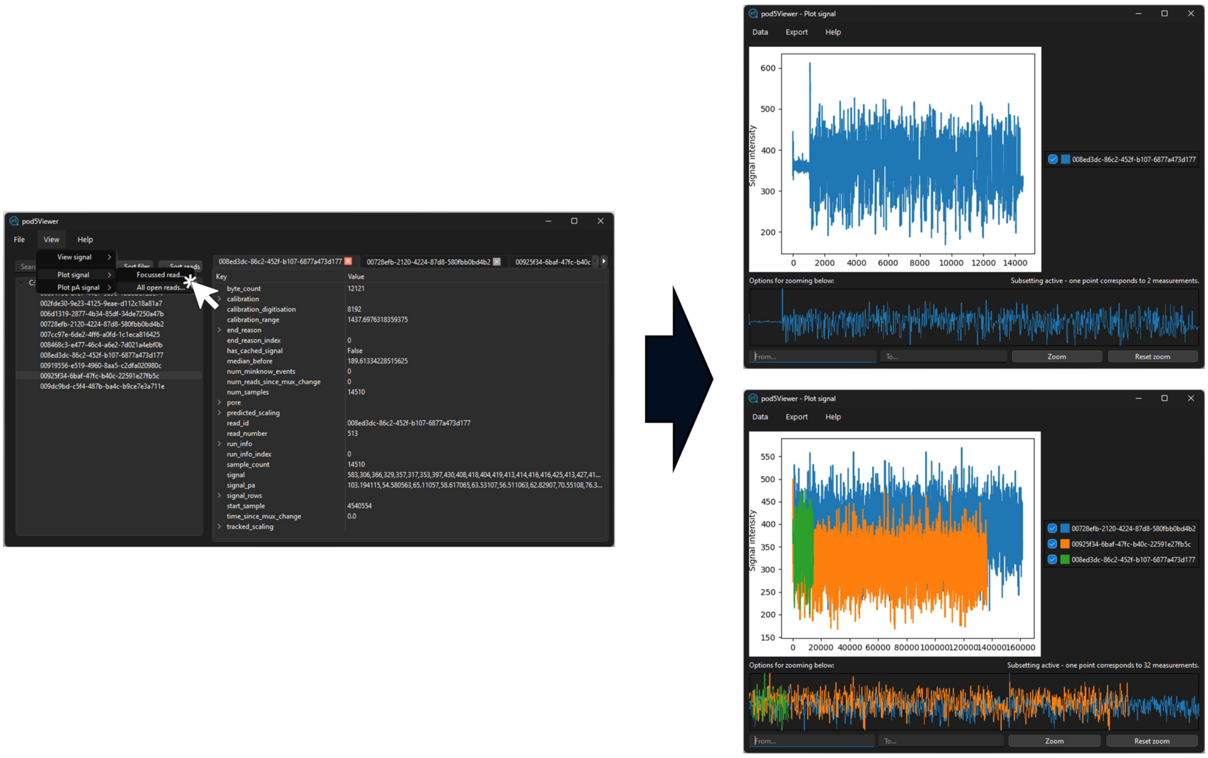

Figure 6 Plotting current measurements. Left: Steps to take for opening the plotting window. Right: Signal plotting window when opening the currently focused read (top) or all opened reads (bottom).

1.2.7 Export functions

Data from one or all opened reads can be exported to JSON format via File > Export all info > Current read... or All opened reads..., respectively (Figure 7, left). Upon selecting one of these options, a file browser opens, where the user selects a path where the exported file(s) should be stored. A file name for a given read is selected from the read ID (Figure 7, right).

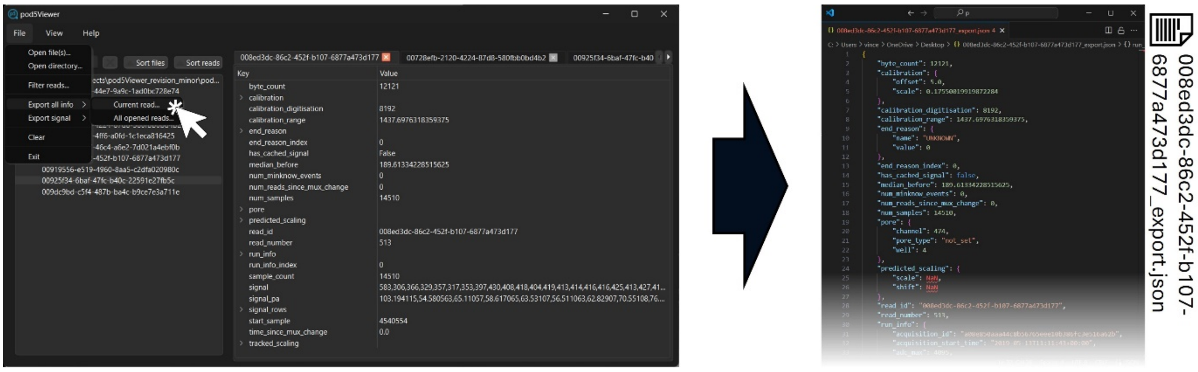

Figure 7 Export read(s) to JSON format. Left: Steps to take for exporting read data to JSON. Right: Head of the exported JSON file.

Alternatively, the signal can be exported isolated from other metadata. This option can be accessed via File > Export signal. Here the measurements – optionally calibrated to pA – can be written to either binary Numpy's *.npy* format or human-readable text format. Exporting can be done for the currently focused read or all opened reads (Figure 8). When exporting to *.npy* format, files can be accessed via the *load* function provided by Numpy[3].

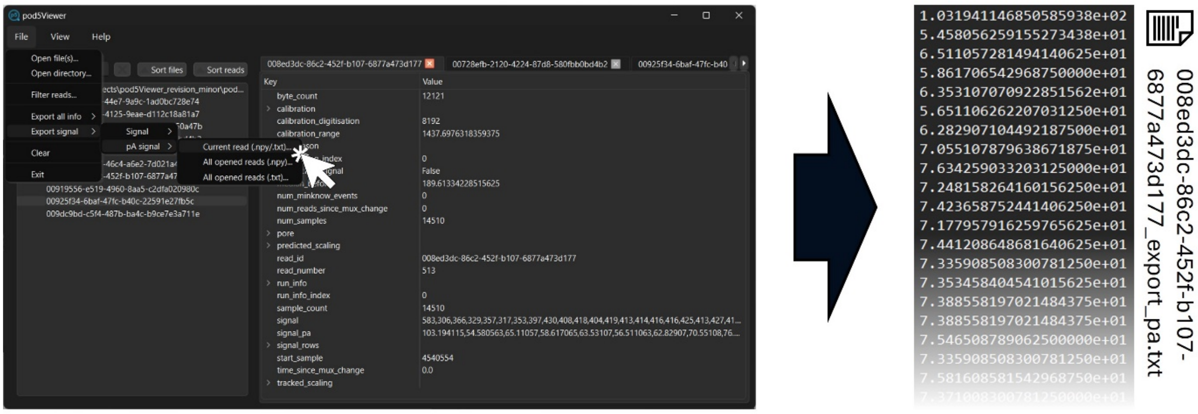

Figure 8 Export signal(s) to binary .npy or human-readable .txt format. Left: Steps to take for exporting a single pA signal. Right: Head of the .txt file after exporting.

1.2.8 Clear window

The entire window can be cleared through File > Clear (Figure 9, left). This removes all loaded elements from the navigator and data view panels (Figure 9, right).

[3] https://numpy.org/doc/stable/reference/generated/numpy.load.html

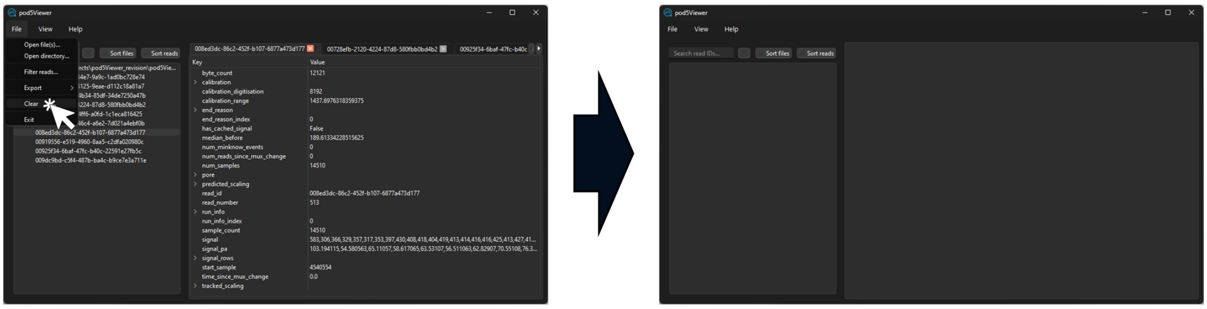

Figure 9 Left: pod5Viewer before clearing with data opened in the data view panel. Right: pod5Viewer after clearing opened files.

1.2.9 Shortcuts

An overview of existing shortcuts can be accessed via Help > Shortcuts. These shortcuts can be split into different groups. The following shortcuts correspond to the file menu:

- Ctrl + O: Open the Open file(s)... menu to select file(s)
- Ctrl + D: Open the Open directory... menu to select a directory
- Ctrl + S: Export the currently focused read
- Ctrl + A: Export all opened reads
- Ctrl + Backspace: Clear the window
- Ctrl + Q: Exit the pod5Viewer

For faster navigation, the following shortcuts are implemented:

- Arrow keys: navigate in the currently focused panel (file navigator or data view panel); *Up* and *down* to move the focus to the next entry; *Right* to expand a nested entry; *Left* to hide an expanded entry
- Tab: switch focus between the file navigator and data view panel
- Ctrl + Tab: Cycle through tabs in the data view panel
- Ctrl + W: Close the currently focused tab in the data view panel

In the signal viewing window implemented shortcuts are:

- Pagedown: Scroll down through chunks in large steps
- Pageup: Scroll up through chunks in large steps
- Arrow Down: Scroll down through chunks in small steps
- Arrow Up: Scroll up through chunks in small steps

Additionally, the menu at the top can be accessed without a mouse through the *Alt* key. After pressing the Alt key, the first letters of the individual menus are underlined. When underlined, pressing the respective key opens the menu which can then be navigated with the arrow keys.

1.3 Backend details

1.3.1 Communication with the pod5 API

Accessing POD5 files is mediated by the Python implementation of the POD5 API by ONT[4]. Selected POD5 files are loaded into a *DatasetReader* object. Upon selecting a reads, it gets selected and opened via the *get_read* method. Contained attributes are transformed into a Python dictionary for subsequent display in the data view panel in the main window of the pod5Viewer.

1.3.2 Details on the plotting functions

1.3.2.1 Plotting backend

For rendering plots, the pod5Viewer uses the QtAgg backend by Matplotlib[5]. This enables all functionalities of the Matplotlib library to be embedded into a Qt canvas. With its fast performance, it allows for dynamic redrawing of a given plot in real-time for dynamic zooming and resizing.

1.3.2.2 Downsampling of measurements

Rendering large amounts of measurements quickly leads to unresponsive behaviour of a GUI, especially for potentially multiple signals with hundreds of thousands of measurements plotted at once. To provide plots that are as responsive as possible, long signals are downsampled to 10000 measurements. This is achieved by splitting the data into 10000 bins of roughly equal size and using the median value of each bin as its representation.

Each time a new range is selected by the user, it is checked if the new interval still contains more than 10000 measurements. If that is the case, downsampling is performed again on the new interval. Otherwise the data is shown in its entirety. This way the extent of the downsampling is dynamically changing, always showing the highest possible resolution of the data, minimizing the loss of information while retaining high responsiveness.

1.3.2.3 Normalization

For normalization the standard score z is calculated for each current measurement x in a signal, where the mean signal intensity μ of the respective read is subtracted and then divided by the standard deviation σ:

$$z = \frac{x - \mu}{\sigma}$$

---

[4] https://github.com/nanoporetech/pod5-file-format
[5] https://matplotlib.org/stable/users/explain/figure/backends.html#interactive-backends

1.4 Possible applications

1.4.1 General data exploration

ONT provides many different options when performing nanopore sequencing. For DNA library preparation alone, kits are available for ligation sequencing with optional barcoding, rapid sequencing with optional barcoding, ultra-long sequencing, rapid PCR barcoding and 16S barcoding (https://nanoporetech.com/products/prepare/dna-library-preparation). Additionally, adaptive sampling is available for most kits. For RNA sequencing on the other hand, both direct RNA and cDNA kits are available, with the latter having the option for both PCR amplification and barcoding (https://nanoporetech.com/products/prepare/rna-library-preparation). After library preparation further options emerge during sequencing via MinION and PromethION flowcells and different sequencing devices for each.

All these options together lead to a wide variety of possible sequencing approaches, which in turn require different approaches in the following bioinformatic processing steps. These include the correct selection of a base-calling model fitting to the chemistry that was used, whether demultiplexing is necessary and whether modified base-calling is an option, among other things. While this information is usually known when the sequencing was performed by the user directly, especially when collaborating with other researchers or using existing data from other publications important information can get lost in the process.

With the pod5Viewer the user can easily double-check important information about the library preparation and sequencing steps, potentially saving a lot of time. Without any previous information the user can extract from a POD5 file the sequencing kit (*run_info* > *sequencing_kit*), sample rate (*run_info* > *sample_rate*), the selected speed (*run_info* > *context_tags* > *selected_speed_bases_per_second*), whether barcoding is enabled (*run_info* > *context_tags* > *barcoding_enabled*) and if so the barcoding kit (*run_info* > *context_tags* > *barcoding_kits*) to specifically determine first processing steps. For example a POD5 file can contain the following information:

- sequencing kit “sqk-nbd114-24”
- sample rate “5000”
- selected speed “400”
- barcoding_enabled “1”

The sequencing kit reveals that R10.4.1 nanopores were used and together with the sample rate, the selected speed and information found in the dorado documentation[6], the user can determine that the latest super accuracy model (dna_r10.4.1_e8.2_400bps_sup@v5.0.0) is fitting for the data. Additionally, the user

[6] https://github.com/nanoporetech/dorado/?tab=readme-ov-file#dna-models

knows that demultiplexing is needed for the sample. Accordingly, the following commands are the first step for processing the information:

dorado basecaller dna_r10.4.1_e8.2_400bps_hac@v5.0.0 *pod5dir* --kit-name SQK-NBD114-24 > *basecalls.bam*

dorado demux --output-dir *barcodedir* --no-classify *basecalls.bam*

1.4.2 Signal plotting

Plotting the signal can be useful when looking for aberrations in the signal of reads of interest (Figure 10). Systematic occurrences of such outliers in the current measurements could indicate issues with a sequencing device or special characteristics of the nucleotide sequences at hand, similar to systematic aberrations in the signal at chemically modified sites. Generally, identifying these outliers could help in identifying causes of increased error rates in specific reads.

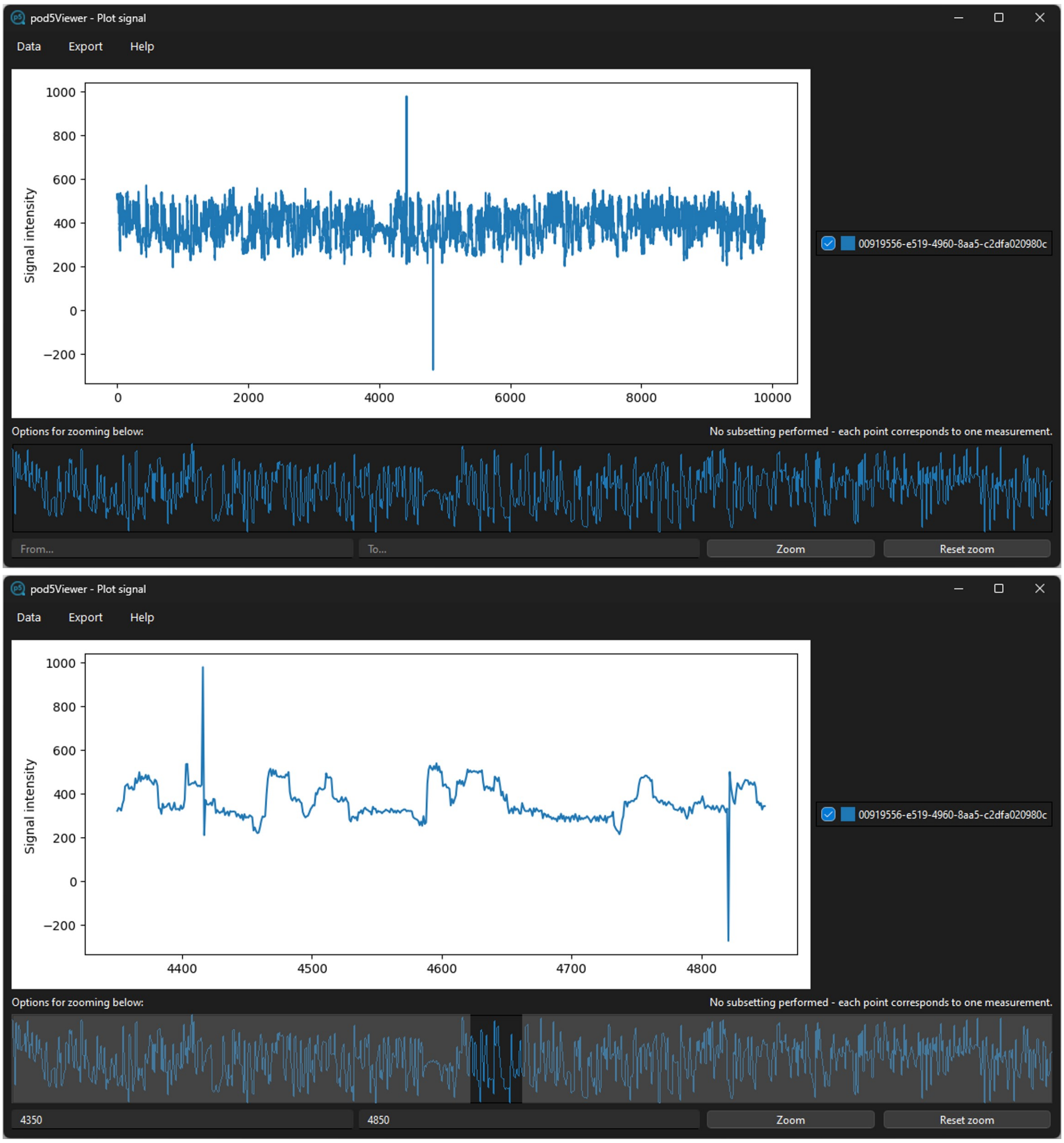

Figure 10 Screenshots of a plotted signal with notable aberrations in the signal in its entirety (top) and zoomed in (bottom). Shown read can be found in test file "multi_fast5_zip_v3.pod5".

1.4.3 Data export

Another possible application includes the downstream analysis after exporting a set of reads to JSON format. The JSON file format is convenient for further processing, as files are easily readable in various programming languages like Python (Code 1), JavaScript (Code 2) and R (Code 3), among others. This enables straight-forward access to the data in various environments.

Code 1 Loading a YAML file using Python

```
import json
with open('exported_read.json', 'r') as file:
	data = json.load(file)
print(data)
```

Code 2 Loading a YAML file using JavaScript

```
const fs = require('fs');

try {

  const fileContents = fs.readFileSync(' exported_read.json', 'utf8');

  const data = JSON.parse(fileContents);

  console.log(data);

} catch (e) {

  console.log(e);

}
```

Code 3 Loading a JSON file using R

```
library(jsonlite)

data <- fromJSON('exported_read.json')

print(data)
```

With data from various reads easily accessible, the user can pursue varied analyses. An obvious approach would be to use the raw signal as input for a machine learning algorithm for a given classification or clustering task. Other metadata from POD5 files could also be included to explore detailed characteristics of the nanopore sequencing data, for example regarding the calibration and scaling.

1.5 Test data

In the Github repository four POD5 files are provided in the test_data directory for testing purposes. These stem from the *pod5-file-format* repository by ONT (https://github.com/nanoporetech/pod5-file-format) where they are freely accessible.
